# British residents’ views about general practice care in France – a telephone survey

**DOI:** 10.1186/1472-6963-13-224

**Published:** 2013-06-18

**Authors:** Nathalie Pelletier-Fleury, Marc Le Vaillant

**Affiliations:** 1 CERMES3 (Centre de Recherche Médecine, Sciences, Santé, Santé Mentale, Société), INSERM (Institut National de la Santé et de la Recherche Médicale) U 988 - UMR 8211, 7, rue Guy Moquet, 94801, Villejuif, France; 2 EHESS (Ecole des Hautes Etudes en Sciences Sociales), F-75244, Paris, France; 3 Université Paris Descartes, F-75005, Paris, France

## Abstract

**Background:**

Many studies have been published over the past decade on patients’ views about the provision of health care. Though there is a high level of migration within the European Union, there are no studies on migrants’ views about the provision of care in the country to which they moved. Given the wide spectrum of primary care in Europe, we hypothesised, without prejudging the outcome, that patients’ confidence in the system they left, used as a proxy of ‘the experience of care’, may influence their preferences regarding specific aspects of care in the host country. The objective of the study was to analyse British migrants’ views on general practice care in France.

**Methods:**

A telephone survey was conducted with a random sample of the adult population of British people residing in France. Participants were 437 women and 423 men, aged 18 and over, who had consulted a general practitioner at least once during the past 12 months. The main outcome measures were the responses to the 23-item Europep questionnaire evaluating different aspects of general practice care, using a five-point answering scale with the extremes labelled as “poor” and “excellent”.

**Results:**

Participants were generally satisfied with the GP care provided. The aspects that were rated the highest were related to the doctor-patient relationship which over 80% of the respondents judged as excellent or very good. Some aspects of the organisation of services received relatively negative evaluations. For instance, “waiting time in the waiting room” was evaluated as excellent or very good by only 40% of the respondents. Twenty seven percent of the respondents were not confident in the National Health Service (NHS) when they were still living in UK. After adjusting for age, sex and number of years of residence in France, the respondents who were not confident in the NHS provided a score of “excellent” significantly more frequently (on 11 out of the 23 aspects of care) than did the patients who were confident in the NHS. Most of these aspects concerned the doctor-patient relationship and information and support during the consultation.

**Conclusions:**

British migrants’ views on general practice care in France varied with the degree of confidence they had in the NHS. This finding is in line with the discussion on whether the ‘experience of care’ influences patient satisfaction. A better understanding of this phenomenon should provide valuable insights to make the services more responsive to the patients.

## Background

Many studies have been published over the past decade on patients’ views about the provision of health care. The aim has been to find ways to make the services more responsive to patients and to the public in general [1]. Patient characteristics, including demographic and cultural factors, have been shown to be predictors of preferences regarding many aspects of primary health care [2]. Comparisons of patients’ views between European countries [3] or between the United States and Commonwealth countries [4] have also been well documented.

Though there is a high level of migration within the European Union, to our knowledge, there are no studies on migrants’ views about the provision of care in the country to which they moved. Given the wide spectrum of primary care in Europe, we hypothesised, without prejudging the outcome, that patients’ confidence in the system they left, which was used in this article as a proxy of ‘the experience of care’ [4], may influence their preferences regarding specific aspects of care in the host country.

In this study, we assess British residents’ views on general practice care in France. We then compare the participants based on the degree of confidence they had in the National Health Service (NHS) when they were still living in the UK.

## Methods

### Sampling design

A telephone survey was conducted with a random sample of the adult population (aged 18 and over) of British people residing in France. The fieldwork was organised by IFOP, a French-based market research institute, using computer-assisted telephone interviewing (random-digit dialling). The target population was identified through a telephone directory using a list of names provided by the Office for National Statistics. The study was conducted in March 2011.

We contacted 4057 individuals. People who were not natives of Great Britain (from the United States, from South Ireland, or from another country) (n=2119), whose main residence was not in France (n=147) and who had never lived in Great Britain before (n=11) were excluded. Of the remaining 1780 eligible participants, 1004 agreed to participate (56%). Among these participants, 144 had not consulted a general practitioner (GP) at least once during the past 12 months and therefore were unable to respond to the questionnaire. Thus, a total of 860 respondents participated in the study.

#### Ethical approval

The survey was approved by the Commission National Informatique et Libertes (http://www.cnil.fr/english/) (number 2010–419, the 16th of November, 2010). This commission is in charge of examining both ethical and anonymity issues of research protocols. There was no possible linkage between the identity of the respondents and their answers to the questionnaire. The participants gave oral consent.

### Questionnaire

The Europep questionnaire was used [5]. The core of the questionnaire is a set of 23 questions evaluating different aspects of general practice care, using a five-point answering scale with the extremes labelled as “poor” and “excellent”. The items are organised into 4 domains: doctor-patient relationship (6 items), information and support (4 items), medical-technical care (5 items) and organisation of service (8 items) [6]. The respondents were asked to evaluate care provided in France within the past 12 months. The interviews were conducted in English. General and demographic data were also collected from the participants.

### Statistical analysis

Reliability analysis was used to verify the internal consistency (Cronbach’s coefficient alpha) within the Europep instrument. The items were dichotomised according to 1 versus 2–5 (excellent versus less than excellent). This procedure is recommended as a standard for Europep users [7]. Frequency distributions were used to describe the participants’ characteristics and their responses to the 23-items questionnaire.

We used chi-square tests to compare their characteristics and responses to the questionnaire according to whether respondents were confident (very/somewhat confident) or not (not very confident/not at all) in the NHS when they were still living in the UK.

The association between scoring an item “excellent” and the degree of confidence in the NHS was also investigated for all items after adjusting for age (18 to 39 years / 40 to 54 / 55 and over), gender (M/F) and number of years of residence in France (2 years or less / 2 to 5 / more than 5). Odds ratios (ORs) and 95% confidence intervals (CIs) were calculated. We used SAS® software.

## Results

### General characteristics

The characteristics of the study population are summarised in Tables 1, 2 and 3. Ninety-five per cent of the respondents were from England. The respondents who reported that they were not confident (not very/not at all) in the NHS (n=234) had characteristics that were similar to those of the respondents who reported that they were confident (very/somewhat) in the NHS (n=626). The only significant difference between the two groups was related to their overall opinion of the French health care system. Respondents who were not confident in the NHS when they were still living in the UK were over-represented among the patients who reported that the current French healthcare system works pretty well (p=0.03).

**Table 1:** Socio-demographic characteristics of the study population (n=860) according to the degree of confidence in the NHS, in %

	**Very confident, somewhat confident**	**Not very or not at all confident**	**P value**	**All study population**
	**n=626**	**n=234**		
Age:				
- 1^st^ quartile	55	54		55
- Median	63	62		63
- 3^rd^ quartile	69	68		69
Mean age in years (SD)	60.4 (11.4)	61.6 (11.2)	0.10	61.2 (11.2)
Gender:				
- male	49.2	44.0		47.8
- female	50.8	56.0	0.18	52.2
Marital status:				
- married or living as married	80.2	85.9		81.7
- single (never been married, divorced or separated)	13.3	9.8		12.4
- widowed	6.5	4.3	0.15	5.9
Age when you left full-time education:				
- 16 years or less	32.2	34.3		32.9
- 17 or 18 years	23.3	27.0		24.4
- 19 years or over	44.5	37.7	0.20	42.7
Professional status:				
- employed full time	17.1	16.2		16.9
- employed part time	8.2	8.1		8.2
- retired	65.3	65.0		65.2
- not employed	9.4	10.7	0.94	9.7
Residence in France:				
- Paris and suburbs	8.3	7.3		8.0
- North-East	3.2	1.3		2.7
- North-West	39.1	44.9		40.7
- South-West	34.7	36.3		35.1
- South-East	14.7	10.2	0.16	13.5
Level of French:				
- excellent	12.5	12.8		12.6
- very good	13.7	13.7		13.7
- good	23.6	21.8		23.1
- fair	30.4	27.8		29.7
- poor	19.8	23.9	0.73	20.9

**Table 2:** Characteristics of the study population regarding health status, insurance coverage, referring doctor, number of consultations with a GP during the past 12 months according to the degree of confidence in the NHS (n=860), (in %)

	**Very confident, somewhat confident**	**Not very or not at all confident**	**P value**	**All study population**
	**n=626**	**n=234**		
Perceived health status:				
- excellent	19.5	17.1		18.8
- very good	31.2	31.6		31.3
- good	34.2	33.8		34.1
- fair, poor	15.1	17.5	0.90	15.8
Existence of any longstanding conditions:				
- one or several longstanding conditions	41.1	40.4		40.8
- not any longstanding condition	58.9	59.6	0.86	59.2
Health insurance coverage:				
- accessing health care via the *assurance maladie* or accessing health care via the *assurance maladie* but temporarily	86.6	89.7		87.4
- accessing health care via a private insurance	8.5	7.7		8.3
- you have not got any insurance coverage	4.9	2.6	0.52	4.3
Registration with a referring physician:				
- yes	96.3	96.8		96.4
- no	3.7	3.2	0.23	3.6
The referring physician is:				
- a GP practicing in solo	47.8	45.1		47.2
- a GP practicing in group practice	48.6	49.6		49.1
- other situation	3.6	5.3	0.58	3.7
Number of consultations with a GP or another specialist (not counting doctors seen when hospitalized) during the past 12 months:				
- 1-2	34.8	28.2		33.0
- 3-4	32.9	39.3		34.7
- 5-6	18.1	20.5		18.7
- 7 or more	14.2	12.0	0.14	13.6

**Table 3:** Characteristics of the study population regarding reasons for moving from UK to France, number of years of residence in France, degree of confidence in the French health care system when moving, actual overview of the French healthcare system according to the degree of confidence in the NHS (n=860)

	**Very confident, somewhat confident**	**Not very or not at all confident in %**	**P value**	**All study population**
	**n=626**	**n=234**		
Reasons for moving from France to UK*:				
- Professional reasons				
- Yes	13.3	10.7	0.2266	12.6
- Family reasons				
- Yes	16.1	13.4	0.2301	15.4
- Retirement				
- Yes	33.6	32.9	0.1409	33.4
Number of years of residence in France:				
- 2 years or less	5.6	5.6		5.6
- 2 to 5 years	24.9	26.9		25.5
- more than 5 years	69.5	67.5	0.8335	68.9
Mean number of years of residence in France (SD)	11.2 (10.0)	10.0 (8.3)	0.1037	10.9 (9.5)
Actual overall view of the French health care system:				
- works pretty well	86.8	93.2		88.7
- fundamental changes are needed to make it work better or need to be completely rebuilt	13.2	6.8	0.0356	11.3

* People can have different reasons (the sum of percentages is more than 100%).

### Responses to the Europep questionnaire

The participants’ responses to the Europe questionnaire are summarised in Table 4. The Cronbach’s coefficient alpha was 0.97. For most of the selected aspects of care (17 out of 23), more than 75% of the respondents believed that the care was very good or excellent. The aspects that were scored the highest pertained to the doctor-patient relationship, which over 80% of the respondents judged as excellent or very good. An exception was the aspect “involving you in decisions about your medical care”, where only 70% gave high scores. It is noteworthy that the aspects pertaining to the organisation of medical practice received significantly lower scores. For instance, “preparing you for what to expect from specialists, hospital care or other care providers”, “being able to talk to the general practitioner on the telephone” and “waiting time in the waiting room” were evaluated as excellent or very good by only 69, 67 and 40% of the respondents, respectively.

**Table 4:** Evaluation of English residents on general practice care in France using the 23-items questionnaire of the Europep (n=860)

**Items**	**Excellent (in %)**	**Very good (in %)**	**Good (in %)**	**Fair (in %)**	**Poor (in %)**
1. Making you feel you have time during consultation (I)	68.62	20.49	7.49	2.34	1.05
2. Showing interest in your personal situation (I)	60.10	23.56	11.54	3.97	0.84
3. Making it easy for you to tell him or her about your problem (I)	60.05	23.52	12.88	2.72	0.83
4. Involving you in decisions about your medical care (I)	53.46	26.42	14.69	4.20	1.23
5. Listening to you (I)	57.41	26.00	13.53	2.35	0.71
6. Keeping your records and data confidential (I)	63.41	23.96	11.07	1.17	0.39
7. Providing quick relief of your symptoms (II)	55.21	27.60	14.55	2.26	0.38
8. Helping you to feel well so that you can perform your normal daily activities (II)	53.43	29.96	13.98	2.25	0.37
9. Thoroughness of the approach to your problems (II)	54.42	27.45	13.60	3.94	0.60
10. Physical examination of you (II)	53.55	30.02	13.85	1.96	0.61
11. Offering you services for preventing diseases (e.g. screening, health checks, immunizations) (II)	48.98	28.71	15.65	4.63	2.04
12. Explaining the purpose of examinations, tests and treatments (III)	47.24	31.91	15.58	4.65	0.63
13. Telling you enough about your symptoms and/or illness (III)	44.60	32.17	16.52	5.96	0.75
14. Helping you deal with emotions related to your health status (III)	42.21	30.25	19.20	6.34	1.99
15. Helping understand why it is important to follow the GP’s advice (III)	42.32	33.94	20.11	2.65	0.98
16. Knowing what has been done or told during previous contacts in the practice (IV)	42.82	30.94	20.86	4.56	0.83
17. Preparing you for what to expect from specialists, hospital care or other care provider (IV)	38.81	29.89	19.56	8.76	2.97
18. The helpfulness of the practice staff (other than the doctor) to you (IV)	41.89	33.28	19.09	4.39	1.35
19. Getting an appointment to suit you (IV)	47.30	29.04	15.69	5.64	2.33
20. Getting through to the practice on telephone (IV)	47.16	31.70	15.98	4.12	1.03
21. Being able to talk to the general practitioner on the telephone (IV)	41.92	25.04	21.67	7.28	4.09
22. Waiting time in the waiting room (IV)	16.04	23.58	30.19	19.58	10.61
23. Providing quick services for urgent health problems (IV)	50.53	30.23	15.11	3.51	0.61

I : domain “doctor-patient relationship”; II: domain “information and support”; III: domain “medical-technical care”; IV: domain “organization of services”.

### Degree of confidence in the NHS and responses to the Europep questionnaire

All aspects of care were more frequently scored as excellent by respondents who were not confident (not very/not at all) in the NHS compared with respondents who were confident (very/somewhat) in the NHS (Table 5). After adjusting for age, sex and number of years of residence in France, the respondents who were not confident in the NHS provided a score of “excellent” significantly more frequently on 11 out of the 23 aspects of care than did the patients who were confident in the NHS (Table 6). These 11 aspects included 4 aspects out of 6 regarding the doctor-patient relationship and 2 out of 4 regarding information and support but only 2 out of 5 regarding medical-technical care and 3 out of 8 regarding the organisation of services.

**Table 5:** Proportions of respondents who scored the 23 items of the Europep “excellent” according to the degree of confidence in the NHS

**Question**	**Domain**	**Very confident, somewhat confident**	**Not very, not at all confident**	**P value**
		**n=626**	**n=234**	
1*	I	66,67	73,82	0,04
2*	I	57,19	67,84	0,01
3	I	59,35	61,90	0,49
4	I	52,05	57,08	0,19
5*	I	54,85	64,22	0,01
6*	I	61,11	69,52	0,03
7	II	53,57	59,46	0,13
8	II	51,81	57,66	0,13
9	II	52,87	58,52	0,14
10*	II	51,43	59,19	0,04
11*	II	46,30	55,77	0,02
12*	III	44,81	53,67	0,02
13	III	42,51	50,00	0,06
14*	III	39,05	49,13	0,02
15	III	40,58	46,77	0,13
16*	IV	40,61	48,73	0,04
17	IV	37,99	40,88	0,49
18	IV	40,05	46,47	0,15
19	IV	45,53	52,02	0,09
20	IV	46,52	48,84	0,56
21	IV	39,55	47,59	0,07
22*	IV	14,40	20,43	0,03
23*	IV	47,55	58,06	0,01

I : domain “doctor-patient relationship”; II: domain “information and support”; III: domain “medical-technical care”; IV: domain “organization of services”.

*items scored significantly more frequently “excellent” by respondents who were not confident in the NHS (not at all / not very) (p<0.05).

**Table 6:** Relation between scoring an item “excellent” with the degree of confidence in the NHS expressed as odds ratios (95% confidence interval)

**Question**	**Domain**	**Unadjusted odds ratios**		**Adjusted odds ratios on age and sex**		**Adjusted odds ratios on age, sex and number of years of residence in France**	
		**Value**	**CI**	**Value**	**CI**	**Value**	**CI**
1*	I	1.41	1.01 1.97	1.43	1.02 2.00	1.43	1.02–2.00
2*	I	1.58	1.15– 2.18	1.61	1.17– 2.23	1.61	1.17–2.23
3	I	1.11	0.82– 1.51	1.13	0.83 – 1.54	1.13	0.83–1.54
4	I	1.23	0.90– 1.66	1.25	0.91– 1.71	1.26	0.92–1.72
5*	I	1.48	1.08– 2.02	1.49	1.09– 2.04	1.50	1.09–2.05
6*	I	1.45	1.03– 2.04	1.48	1.05 – 2.08	1.48	1.05 – 2.09
7	II	1.27	0.93– 1.74	1.28	0.93– 1.75	1.27	0.93–1.75
8	II	1.27	0.93– 1.73	1.28	0.93– 1.75	1.28	0.93–1.75
9	II	1.26	0.93– 1.71	1.28	0.94– 1.74	1.28	0.94 – 1.75
10*	II	1.37	1.00– 1.88	1.39	1.02 – 1.90	1.39	1.02–1.91
11*	II	1.46	1.06– 2.02	1.47	1.06– 2.03	1.47	1.06–2.03
12*	III	1.43	1.04– 1.96	1.43	1.05– 1.96	1.44	1.05– 1.97
13	III	1.35	0.99– 1.85	1.36	1.00– 1.86	1.37	1.01–1.88
14*	III	1.51	1.05– 2.17	1.52	1.06– 2.19	1.53	1.06–2.20
15	III	1.29	0.93– 1.79	1.30	0.93– 1.81	1.31	0.94–1.82
16*	IV	1.39	1.00– 1.93	1.41	1.02– 1.97	1.42	1.02–1.98
17	IV	1.13	0.79– 1.60	1.14	0.80 – 1.62	1.14	0.80–1.62
18	IV	1.30	0.91 – 1.86	1.29	0.90– 1.86	1.29	0.90–1.85
19	IV	1.30	0.95 – 1.77	1.33	0.98– 1.8	1.33	0.98–1.82
20	IV	1.1	0.80– 1.51	1.10	0.80– 1.51	1.10	0.80–1.51
21	IV	1.39	0.96– 2.00	1.38	0.95– 1.99	1.38	0.95–1.99
22*	IV	1.53	1.03– 2.26	1.59	1.07– 2.36	1.60	1.08–2.38
23*	IV	1.53	1.08– 2.15	1.51	1.07– 2.13	1.52	1.07–2.14

I : domain “doctor-patient relationship”; II: domain “information and support”; III: domain “medical-technical care”; IV: domain “organization of services”.

* items scored significantly more frequently “excellent” by respondents who were not confident in the NHS (not at all / not very).

## Discussion

### Responses to the Europep questionnaire

We observed that British people living in France were generally satisfied with the general practice care provided. The aspects that were rated the highest were related to the doctor-patient relationship. Some aspects of the organisation of services, such as “preparing you for what to expect from specialists, hospital care or other care providers”, “being able to talk to the general practitioner on the telephone” and “waiting time in the waiting room” received relatively negative evaluations. This first result is consistent with a previous report of Grol et al. [3] who compared the opinions of patients in various European countries and found that “patients are generally very positive about the care provided, but improvements in practice management in some countries are requested”.

### Degree of confidence in the NHS and responses to the Europep questionnaire

The main result of our work was to show that a favourable opinion on certain aspects of general practice care in France was independently and positively associated with the lack of confidence (not very/not at all) in the NHS. We should say again that this variable was used as a proxy of ‘the experience of care’ as it is usually the case in the literature [4]. Most of these aspects concerned the doctor-patient relationship and information and support during the consultation. Few of these favorable opinions were related to the organization of practice. In our sample, 27% of the respondents were not confident in the NHS before moving to France. This figure is close to the one found in a recent survey on adults’ views of care systems overall in five Commonwealth countries in which 23% of respondents in the UK reported that they were not (or not at all) confident in the NHS [4]. The design of our study does not enable us to draw any causal link between a favourable opinion on certain aspects of general practice care in France and a lack of confidence in the British health system in general, and it was not the purpose of the study. However, the association that we have highlighted raises our curiosity because it appeared for all of the care items, even if it was not always statistically significant. It can be argued that results could have been different if we had made a comparative analysis by domains. However, as far as we know, the issue of the unidimensionality of the domains is debated and it remains unclear how to use the proposed domains [8]. This result is quite interesting since the issue of to what extent patient experience explains satisfaction with the health-care system is currently debated. Although it is more balanced in the study by Bleich et al. [9], the literature suggests that much of the remaining variation in health system satisfaction after adjusting for factors commonly used to measure the concept is a reflection of patient experience [10-12].

### Limitations

This study has got some limitations. Our sample was not representative of all British citizens living in France because individuals under the age of 40 were under-represented. Individuals in this age group were difficult to reach by telephone, particularly during the day, despite efforts to call them between 7pm and 9pm. However, this under-representation was not an obstacle with regard to the objectives of the study, which focused on the users of the health care system over the past 12 years, not many of whom are in this age group. A second limitation concerns the degree of confidence in the NHS. This was not a degree of confidence in the NHS expressed at the same time t for all the respondents, since they migrated at different times, and the NHS has been reformed over the past ten years. This limitation emphasises that one must be cautious about finding any causal link between a lack of confidence in the NHS and a favourable opinion of certain aspects of health care in France. A third limitation concerns a potential recall bias. Actually, almost 70% of the respondents left the UK at least 5 years ago. However, we did not ask respondents to recall precise details about the NHS, just to rate whether they were confident or not in the NHS when they were still living in the UK which did not represent a very important memory effort. Especially as these individuals had for most of them (three quarters of the individuals being older than 55 years old) a long experience of care provided by GPs before moving to France and had probably the time to form their opinion. Finally, due to time constraints, we did not ask people about their living conditions in the UK. This factor could have been linked to their degree of confidence in the NHS and could therefore influence the interpretation of our results.

## Conclusions

British migrants’ views on general practice care in France varied with the degree of confidence they had in the NHS when they were still living in the UK. This finding is in line with the discussion on whether the experience of care influences patient satisfaction. A better understanding of this phenomenon should provide valuable insights to make the services more responsive to the patients.

## Consent

Written informed consent was obtained from the patient for publication of this report.

## Competing interests

The authors declare that they have no competing interests.

## Authors’ contributions

NPF conceived of the study, participated in its design and coordination, and drafted the manuscript. MLV participated in the design of the manuscript, performed the statistical analysis and participated in the draft of the manuscript. Both authors read an approved the final manuscript.

## Pre-publication history

The pre-publication history for this paper can be accessed here:

http://www.biomedcentral.com/1472-6963/13/224/prepub
